# Honey bee and native solitary bee foraging behavior in a crop with dimorphic parental lines

**DOI:** 10.1371/journal.pone.0223865

**Published:** 2019-10-11

**Authors:** María Cecilia Estravis Barcala, Florencia Palottini, Walter Marcelo Farina

**Affiliations:** 1 Universidad de Buenos Aires, Facultad de Ciencias Exactas y Naturales, Departamento de Biodiversidad y Biología Experimental, Laboratorio de Insectos Sociales, Buenos Aires, Argentina; 2 CONICET-Universidad de Buenos Aires, Instituto de Fisiología, Biología Molecular y Neurociencias (IFIBYNE), Buenos Aires, Argentina; University of California San Diego, UNITED STATES

## Abstract

Insect pollination is issential for hybrid seed production systems, among which, introduced and native bees are the primary pollinating agents transferring pollen from male fertile (MF) to male sterile (MS) lines. On a highly dimorphic sunflower (*Helianthus annuus*) crop, we assessed the foraging behavior of solitary *Melissodes* bees and honey bees *Apis mellifera*. We found that *Melissodes* spp. were dominant in and showed fidelity to MF plants, gathering sunflower pollen efficiently throughout the day. In contrast, honey bees dominated on MS lines, mostly gathered nectar and exhibited high floral constancy, even after interacting with a second visitor. Also, honey bees carried sunflower pollen on their bodies while visiting MS inflorescences. This study highlights the need for a thorough understanding of the factors involved in a pollinator-dependent agroecosystem crop to assess the contribution of native bees on pollination of crops which offer resources spatially separated in two highly dimorphic parental lines.

## Introduction

Although the honey bee *Apis mellifera* L. is the most adaptable and commonly applied managed pollinator to enhance the production of different crops, this species is not the only insect that pollinates plants of commercial value [1, 2]. Wild insect pollinators other than honey bees have been recently recognized for their role in improving and stabilizing crop-pollination services, because fruit set significantly increases with visitation rate and species richness of wild pollinators, mainly native solitary bees [3–5]. Moreover, Brosi and Briggs [6] demonstrated that the ecosystem functional roles of species are dynamic and can be in fact shaped via interspecific interactions. They showed that the loss of a single species can reduce pollination functioning of the system, even though other potentially efficient pollinators were present.

In the case of sunflower, *Helianthus annuus* L., a crop grown worldwide for the production of oil or for direct consumption of seeds, its reliance on animal pollination varies depending on the degree of self-compatibility of such oilseed or confection cultivars [7–9]. However, insect pollination is issential for hybrid seed production systems, among which, introduced and native bees are the primary pollinating agents transferring pollen from male fertile (MF) to male sterile (MS) lines. MF plants (whose inflorescences offer both nectar and pollen) are sown in rows, alternated with a higher proportion of MS sunflowers (which offer only nectar). In this agroecosystem, honey bees have been shown to carry more sunflower pollen on their bodies in fields where wild bees were more abundant [10], denoting that non-*Apis* bees could augment the pollination efficiency of *Apis mellifera*. In addition, subsequent studies, both in commercial and hybrid sunflowers, revealed that non-*Apis* bees indirectly contributed to crop pollination, as a consequence of their interactions with honey bees which promoted movements of the latter between inflorescences. These interspecific interactions altered honey bees foraging behavior, increasing pollen flow and thus, positively affecting pollination success [8, 11].

Wild bees of the genus *Melissodes* are important sunflower pollinators in the whole American continent. Their presence has been reported both in commercial sunflower fields [9, 12, 13] as well as in seed-production systems [10, 14–16]. These solitary bees collect both pollen and nectar during single foraging bouts and they have been more frequently found foraging on MF than MS sunflowers. However, the difference in their abundance among parental lines seems to depend on the planting schemes (MF:MS rows ratio) and the density of bees gathering pollen present in the crop [10].

In addition to variation in pollinator community composition and pollen availability, bees foraging behavior can be modified by differences in floral morphology [17, 18]. Sunflower breeding programs aim to develop high-yielding varieties, and regularly measure certain morphological characters such as plant height and head size which contribute to seed yield [19, 20]. These morphological traits, which influence the attractivity to pollinators, have been shown to vary between parental lines, remarkably in some genotypes. Such phenotypic variation affected floral constancy of the honey bee *Apis mellifera* in these crops, resulting in a lower percentage of honey bees moving from MF to MS sunflowers with increasing dimorphism between parental lines [21]. As a consequence, increased fidelity to a parental line can translate into less pollen transferred from MF to MS plants, and therefore, can negatively impact the crop yield.

Although Mallinger and coworkers [9] reported the benefits of native wild bees pollinators, including *Melissodes* spp., for confection sunflower yields across the Great Plains, such cultivars do not involve dimorphism between parental lines. The benefits of diverse wild bees assemblages may differ with both variable attractiveness of the hybrids, in particular in crops for seed production, as well as with changing pollinator communities. Such variation in the pollinators foraging behavior in highly dimorphic hybrids, however, has not been previously documented.

In the present study, we assessed the foraging efficiency of both wild *Melissodes* spp and honey bees in pollinating sunflowers crop for hybrid seed production. Specifically, in a first goal, we described parental sunflower morphology in a highly dimorphic system and evaluated the spatial distribution of both populations of bees and their foraging behavior (i.e. floral constancy and exploited resources) across parental lines within the crop. Secondly, we addressed whether their behavior was modified by the intra or interspecific interactions with a second floral visitor. Thirdly, we addressed whether honey bees and other visitors carried sunflower pollen on their bodies while visiting MS lines to evaluate potential transfer of pollen during foraging bouts.

## Materials and methods

### Study site and animals

Field and behavioral studies were performed during the sunflower (*Helianthus annuus*) blooming season in 2017, in a plantation for hybrid seed production (oilseed cultivar). The field (55 ha) was located near Coronel Suárez (37° 58′ 31″ S, 61° 35′ 45″ W), Pampean region, Argentina. The owner of the land, “Pinto y Girones SRL”, gave permission to conduct the study on this site. All variables considered throughout the present study were evaluated on both parental lines of a single sunflower cultivar. The arrangement of the male fertile (MF) and male sterile (MS) lines consisted of two MF lines every eight MS ones, alternated in this proportion throughout the field width. The distance between a sunflower line and its adjacent row was of 50 cm.

A total of 166 colonies of European honeybees (*A*. *mellifera*) with a mated queen, three or four frames of capped brood, food reserves, and about 20,000 individuals were located around the field mentioned above. Ten-frame Langstroth hives were set in groups of 12 or 24 hives each, so that the density of the colonies achieved was of three hives per hectare, which is the suggested stocking rate for sunflower seed production [22]. Beekeepers were informed about the study and provided consent for honey bees manipulation. With respect to *Melissodes* spp., the present study was carried out within its reported natural distribution [12, 13].

### Parental morphology and blooming period

In order to verify the high dimorphism between both parental lines, we measured the height and capitulum diameter of 32 MS and 32 MF plants. The capitulum diameter measurement comprised the whole disc, from a border to the other crossing the center of the capitula.

To assess the phenology of each parental line, the number of floral units at anthesis (*i*.*e*. inflorescences exhibiting at least 30% of the disc flowers with an open corolla) was recorded in a fixed area of 2 m^2^, according to the protocol described by Vaissière and collaborators [23]. This was carried out during January, at the same time as the recording of the abundance of both bee populations in order to assess the total amount of resources (nectar and pollen) available on the study field.

In the agriculture setting considered in this study, MS lines bloomed earlier than MF lines so that the availability of fresh pollen throughout the season was ensured. The quantification of both bee populations was carried out at the beginning of the MF plants flowering period, which occurs during the MS line full bloom.

### Abundance of *Apis mellifera* and *Melissodes* spp. on sunflower parental lines

In order to quantify both bee populations on each parental line, we recorded the number of visitors per 100 open flowering units (ofu) [23] present along transects in between the field rows, considering both parental lines (48 transects in MS lines and 47 MF lines).

To corroborate the identity of wild bees, voucher specimens were collected and later identified and deposited at the Museo Argentino de Ciencias Naturales (MACN, Buenos Aires, Argentina).

### Foraging behavior of *Apis mellifera and Melissodes* spp.

#### Number of inflorescences visited and type of resources exploited

We studied the foraging behavior of the *A*. *mellifera* and *Melissodes* spp. recording the number of inflorescences visited, the parental line and the type of resources exploited. We categorized bees with pollen in the corbiculae as “pollen-collecting bees” and individuals extending their proboscis and without pollen in the corbiculae as “nectar-collecting bees”. To include both pollen and nectar foragers, we made the observations of the visitors’ behavior on the inflorescences between 8:30 and 18:30 h. Records consisted of monitoring individual bees from the moment they landed on a sunflower capitulum until the observer lost sight of the focal bee.

#### Movement of bees and transfer of pollen between parental lines

Given the fact that hybrid sunflower seed production depends on bees transferring pollen from MF to MS inflorescences, we evaluated the floral constancy of *Apis mellifera* and *Melissodes* spp. while foraging on MF sunflowers. To calculate the percentage of bees that showed constancy on MF lines, we considered the following: number of bees foraging constantly on MF plants *100 / [(number of bees foraging constantly on MF) + (number of bees switching from MF to MS)]. Additionally, the mentioned variables were registered for bees foraging on MS lines.

To address the effect of interactions with a second floral visitor, we applied the methodology described in Greenleaf and Kremen [11]. A focal individual foraging on sunflower (both on MS and MF lines) was observed for a maximum of 10 min until another visitor landed on the same inflorescence. During this interaction, we registered the identity of the second visitor and we recorded the behavior of the focal visitor, whether it remained on the same capitulum, or it moved to another sunflower, distinguishing in this case if it visited another inflorescence of the same parental line, or it switched to the other parental line. If a third visitor landed on the sunflower head, or if the focal visitor flew off the initial inflorescence and was lost before it landed on another inflorescence, the observation was discarded.

Finally, we registered the occurrence of sunflower pollen grains adhered to the bodies of 100 floral visitors captured foraging on MS plants either adjacent to MF plants, or two, three and four rows away (50, 100, 150 and 200 cm away from MF rows). All captured visitors were frozen singly for later examination.

At the laboratory, we observed the entire body surface under a stereomicroscope (Leica MZ8) and registered the presence/absence of sunflower pollen grains. We then poured a drop of distilled water on a slide and rolled a single specimen on it, so that its entire body surface would come into contact with the liquid. We added a coverslip and observed the pollen grains under microscope (Labomed CXR III microscope). In order to verify the identity of the pollen, we then compared the sample obtained from the body surface with one obtained from anthers of MF flowers [24].

### Statistics

All statistical tests were performed with R v3.5.1 [25]. Morphological variables of sunflower inflorescences (plant height and capitulum diameter) were assessed by means of generalized linear model (GLM), following a Gaussian error distribution and using the *glm* function of the lme4 package [26]. In this case, we considered the parental line (two-level factor) as a fixed effect. To account for heteroscedasticity in the capitulum diameter between parental lines, a generalized least squares regression (GLS, ‘varIdent’ function in the nlme package [27]) was run. In addition, to test for differences among bee density, we proposed a GLMM with a poisson error distribution, with visitor (2 levels: A, *mellifera*, *Melissodes* spp.) and parental line (2 levels: MS or MF) included as fixed effects. We used the function *glm* of the lme4 package [26]. In this model we included the percentage of blooming as an offset, to correct the number of events for an estimate of population size, and the transect as random factor. We included a two-way interaction between parental line and visitor and we conducted *post hoc* comparisons across parental lines with function ‘emmeans’ [28].

To analyze the foraging behavior of both visitors, we proposed two GLMMs to test the influence of parental line and visitor (fixed factors) on (1) the number of flowering units visited per observation (response variable); and (2) the type of resources exploited (response variable). For the first response variable we considered a negative binomial error distribution to account for the overdispersion of the data with the function *glm*.*nb* of the MASS package [29], including the observation duration as an *offset* and the transect as random factor. The other response variables followed a binomial error distribution. We additionally included a two-way interaction between visitor and parental line, in order to assess variation in bees foraging behavior across sunflower parental lines. We did not include three-way interactions with the time of day (morning or afternoon), as sample sizes per parental line and/or per time of day were too small to analyze statistically. To assess differences in resource foraging during the day by both visitor groups, a subset of the total data including only MF plants was used for analysis. Each observation was assigned to two time periods: Morning (8:30–13:00 h) or Afternoon (14:00–18:30 h), and then analysed using a binomial error distribution, including a two-way interaction between visitor and time of day.

Models in general were simplified as follows: significance of the different terms was tested starting from the higher-order terms model using *anova* function to compare between models [30]. Non-significant terms (p > 0.05) were removed (S1 Table).

On the other hand, we analysed the floral constancy and the behavior after interacting with a second visitor with Fisher’s exact test [31] and the presence of pollen grains on their bodies with a chi-square homogeneity test [32].

## Results

### Parental morphology and blooming period

In order to assess the spatial distribution of honey bees and *Melissodes* bees on each parental line in the sunflower hybrid studied, we evaluated the dimorphism between male sterile and male fertile plants. As expected, the selected cultivar was highly dimorphic with significant differences in the plant height (Minimal adequate model: Plant height ~ parental line, p < 0.001) and the capitulum diameter (Minimal adequate model: Capitulum diameter ~ parental line, p < 0.001). Male sterile plants were smaller than male fertile inflorescences (MS height = 84.8 ± 1.4 cm, N = 32; MF height = 100.6 ± 1.3 cm, N = 32) and their capitulum diameter resulted bigger (MS diameter = 117.19 ± 3.4 mm, N = 32; MF diameter = 74.22 ± 1.6 mm, N = 32).

Both parental lines differed in the percentage of blooming during the experimental period (N = 53 quadrats /parental line). While less than 20% of MF plants were at anthesis by the end of the study, MS plants surpassed 50% blooming on the first day, reaching 81% by the third day. It is worth mentioning that during the study period a total of 435 MF and 571 MS sampled inflorescences were at anthesis, providing enough resources for pollinators to visit them.

### Abundance of *Apis mellifera* and *Melissodes* spp. on sunflower parental lines

To quantify both bee populations on each parental line, all diurnal sunflower visitors were surveyed (S2 Table). Hymenopterans were the most abundant visitors: *A*. *mellifera* (66% of a total of 2,515 surveyed insects), *Melissodes tintinnans* Holmberg and *Melissodes rufithorax* Brèthes (17%). These three species (family Apidae) were present on MS and MF plants, but differed on their spatial distribution between parental lines. While *A*. *mellifera* was dominant on MS sunflowers (87%), *Melissodes* spp. were most abundant on MF inflorescences (52%), followed by honey bees in second place (20%). In the case of *Melissodes* spp., the majority of specimens were female bees, but we also recorded the presence of males (412 females and 20 males). When taking into consideration the sex distribution of *Melissodes* bees, females represented the 85% of these native bees in MS sunflower and the 96% in MF plants.

**Fig 1:**
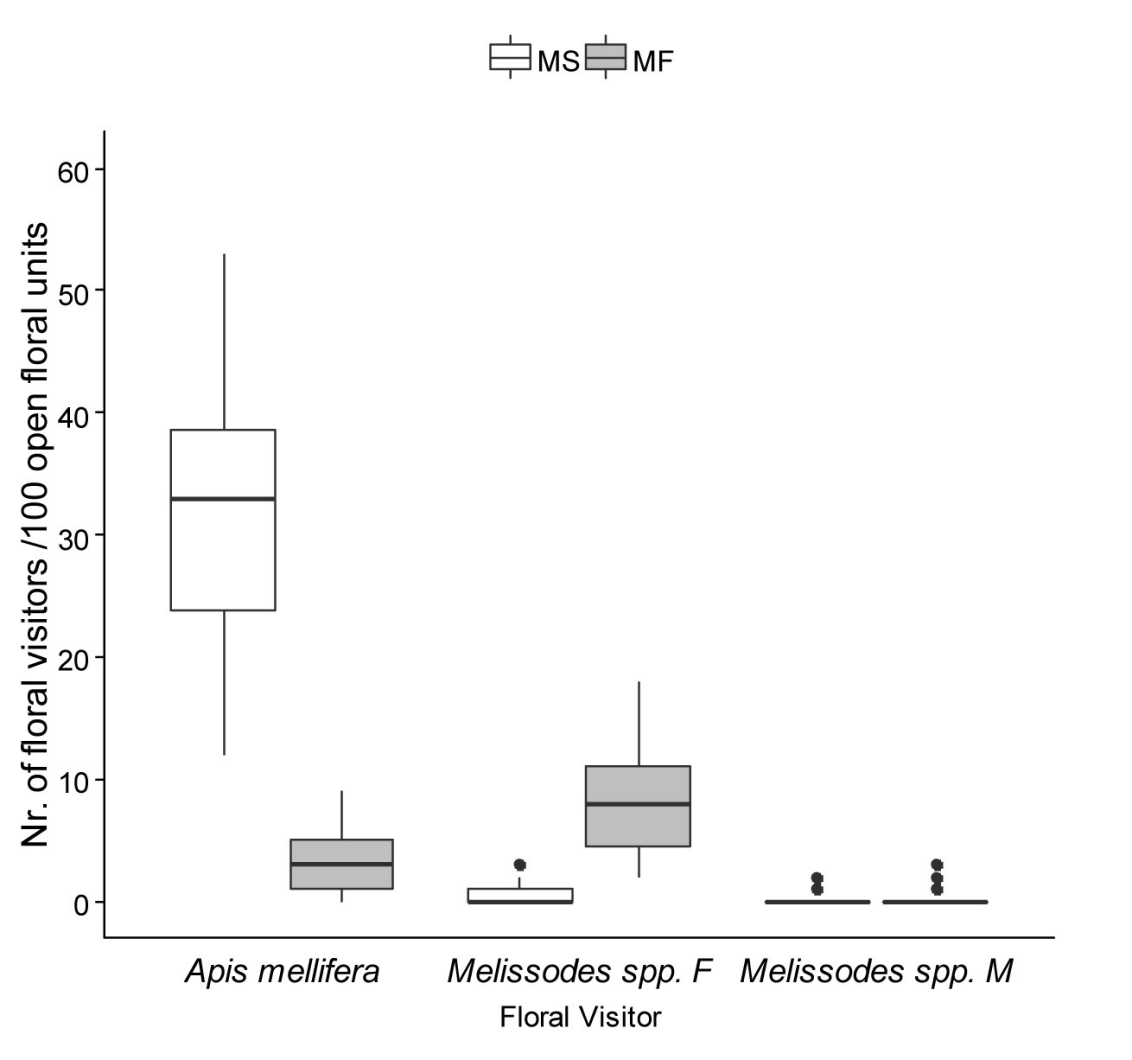
Abundance of main visitors foraging on male sterile (MS) and male fertile (MF) sunflower, including *Apis mellifera*, female *Melissodes* spp (*Melissodes* spp. F) and male *Melissodes* spp. (*Melissodes* spp. M). Number of individuals /100 open floral units were recorded along 48 and 47 transects corresponding to MS and MF rows, respectively. Honeybees were the main visitors on MS lines, while female *Melissodes* spp. were more abundant on MF ones. (Minimal adequate model: Density ~ visitor * parental line + (1|transect) + offset (log (blooming), p < 0.001). Boxplot shows the median and interquartile range (IQR), with whiskers showing the maximum value within 1.5 IQR, and individual points mark values outside this range.

The statistical analysis revealed significant differences in the density of the mentioned bee populations along transects of both parental lines (Fig 1). The number of individuals foraging on 100 open flowering units (ofu) differed significantly between the main groups of visitors, between MS and MF rows, with a significant visitor by parental line interaction (Chisq = 1207.5, p < 0.001, Minimal adequate model: density ~ visitor * parental line + (1|transect) + offset (log (blooming))). On average per transect, the number of honey bees foraging on MS sunflowers (31.5 ± 1.5 indiv./100 ofu, N = 48) showed a nine-fold increase when compared to the ones on MF plants (3.4 ± 0.4 indiv./100 ofu, N = 47; estimated marginal means contrast, p = 0.0001). On the other hand, *Melissodes* spp. were 16 times more frequently found foraging on MF than on MS inflorescences (8.6 ± 0.6 indiv./100 ofu, N = 47; 0.5 ± 0.1 indiv./100 ofu, N = 48, respectively), more than doubling the population of *Apis mellifera* visiting MF sunflowers (estimated marginal means contrast, p < 0.0001).

### Foraging behavior of main visitors

#### Number of inflorescences visited and type of resources exploited

**Fig 2:**
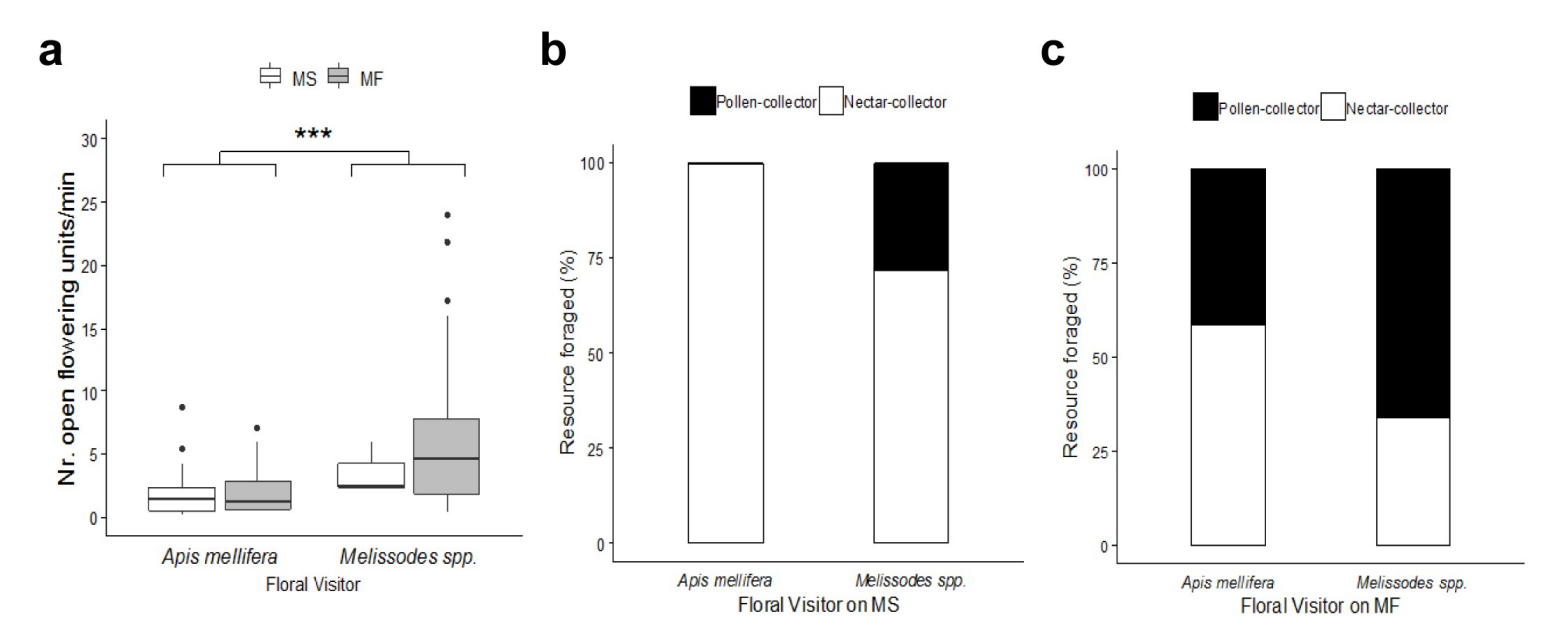
Foraging behavior of *Apis mellifera* and *Melissodes* spp on sunflower inflorescences. (a) Number of floral units visited per min by both groups of visitors, considering male sterile (MS) and male fertile (MF) lines. Observations began once a bee landed on a capitulum and continued along its successive visits to different inflorescences, until observers lost sight of the focal bee. *Melissodes* spp. visited a higher number of inflorescences per minute than honey bees, regardless the parental line. (Minimal adequate model: Nr. floral units ~ visitor + offset(log(time)); *A*. *mellifera*: N = 24 on MS, N = 37 on MF; *Melissodes* spp.: N = 3 on MS, N = 54 on MF). Asterisks indicate statistical differences (***, p < 0.001). Boxplot shows the median and interquartile range (IQR), with whiskers showing the maximum value within 1.5 IQR, and individual points mark values outside this range. Resources foraged by both groups of visitors on (b) MS and (c) on MF parental lines. On MS flowers, *Apis mellifera* almost exclusively foraged for nectar, while 28% of *Melissodes* spp. carried pollen loads (Minimal adequate model: Resource ~ visitor * parental line; estimated marginal means contrast, z ratio = -7.467, p < 0.0001; *A*. *mellifera*, N = 1536; *Melissodes* spp., N = 25). On MF inflorescences, while most honey bees collected nectar, the majority of *Melissodes* bees carried pollen loads (estimated marginal means, z ratio = -5.776, p < 0.0001; *A*. *mellifera*, N = 196; *Melissodes* spp., N = 448).

When we analysed the number of inflorescences visited of each parental line, differences between *A*. *mellifera* and *Melissodes* spp. were found (Fig 2A). *Melissodes* bees visited a significantly higher number of inflorescences per minute (*Melissodes* spp.: 3.9 ± 0.5 [0.2–16.4] ofu/min, N = 57; *A*. *mellifera*: 1.4 ± 0.2 [0.1–7.5] ofu/min, N = 61). Even though the visitation rate was not modified by the parental line, given the fact that the spatial distribution of *Melissodes* bees was biased towards MF sunflowers, as previously mentioned, only few specimens of this visitor were found foraging on such parental line (S1 Table, Minimal adequate model: Nr. ofu ~ visitor + offset(log(time)), p < 0,001).

Concerning the issue of foraging preferences, we found that both visitors differed significantly in the resources exploited, with a significant visitor by parental line interaction (LRT_1, 2201_ = 30.752, p < 0.001, S1 Table, Minimal adequate model: Resource ~ visitor * parental line, Fig 2B and 2C). As for MS lines, while honey bees foraged almost exclusively for nectar, a 28% of *Melissodes* spp. carried corbicular pollen (Fig 2B; estimated marginal means contrast, z ratio = -7.467, p < 0.0001). No honey bees carrying pollen loads were observed licking nectar on MS inflorescences.

On the other hand, on MF rows most bees of both visitor groups foraged for pollen, but this preference was more evident in *Melissodes* spp (Fig 2C; estimated marginal means, z ratio = -5.776, p < 0.0001). The analysis of resources foraged on this parental line taking into account the time of day, revelead that the majority of *Melissodes* bees carried pollen throughout the day (S1 Table, Minimal adequate model: Resource on MF ~ visitor + time of day, p < 0.0001).

#### Movement of bees and transfer of pollen between parental lines

**Fig 3:**
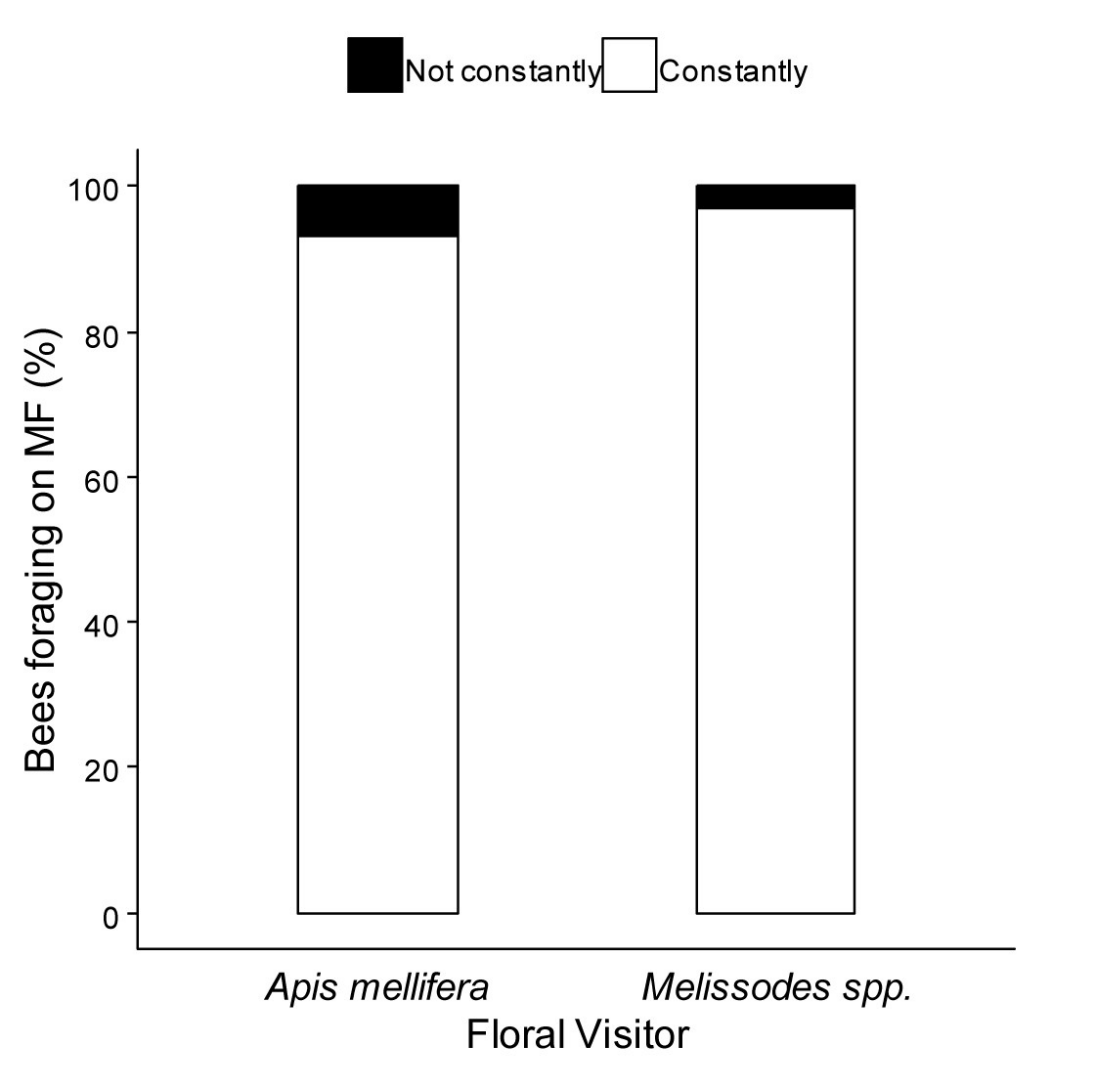
Floral constancy of *Apis* and *Melissodes* bees on male fertile (MF) plants. The percentage of specimens foraging exclusively on the MF parental line is represented with white (constantly). Black bars represent the percentage of visitors switching from MF to male sterile (MS) plants (not constantly). Both visitors exhibited floral constancy (Fisher’s exact test, p = 0.5859; *A*. *mellifera*, N = 29; *Melissodes* spp., N = 35).

When we evaluated the potential transfer of pollen between parental lines, we found that *A*. *mellifera* and *Melissodes* spp behaved similarly in respect to their floral constancy (Fig 3). Specimens showed a noticeable preference to forage on MF rows (Fisher’s exact test, p = 0.5859; *A*. *mellifera*, N = 29; *Melissodes* spp., N = 35). All individuals followed while foraging on MS inflorescences continued to visit the same parental (N = 21, *A*. *mellifera*).

Additionally, the interaction with a second visitor (*A*. *mellifera* or *Melissodes* spp.) did not enhance movement of the focal bee to another floral unit. The majority of the honey bees remained foraging on the same inflorescence and only 20.7% of 29 interactions resulted in the movement to another one. In turn, in the case of *Melissodes* spp. (N = 10) more than half of the individuals (60%) moved to another inflorescence (Fisher’s exact test, p = 0.0427). Moreover, those individuals that moved to another sunflower head did not switch between parental lines. On MS sunflowers, almost all honey bees presented intraspecific interactions (92%, N = 12); however, on the other parental line, the majority of *Apis mellifera* experienced interspecific ones (76%, N = 17) and still continued to forage on the same capitulum afterwards (85%). No interactions of *Melissodes* spp. on MS sunflowers were observed during 120-minute survey, as expected due to their differential distribution by parental lines.

**Fig 4:**
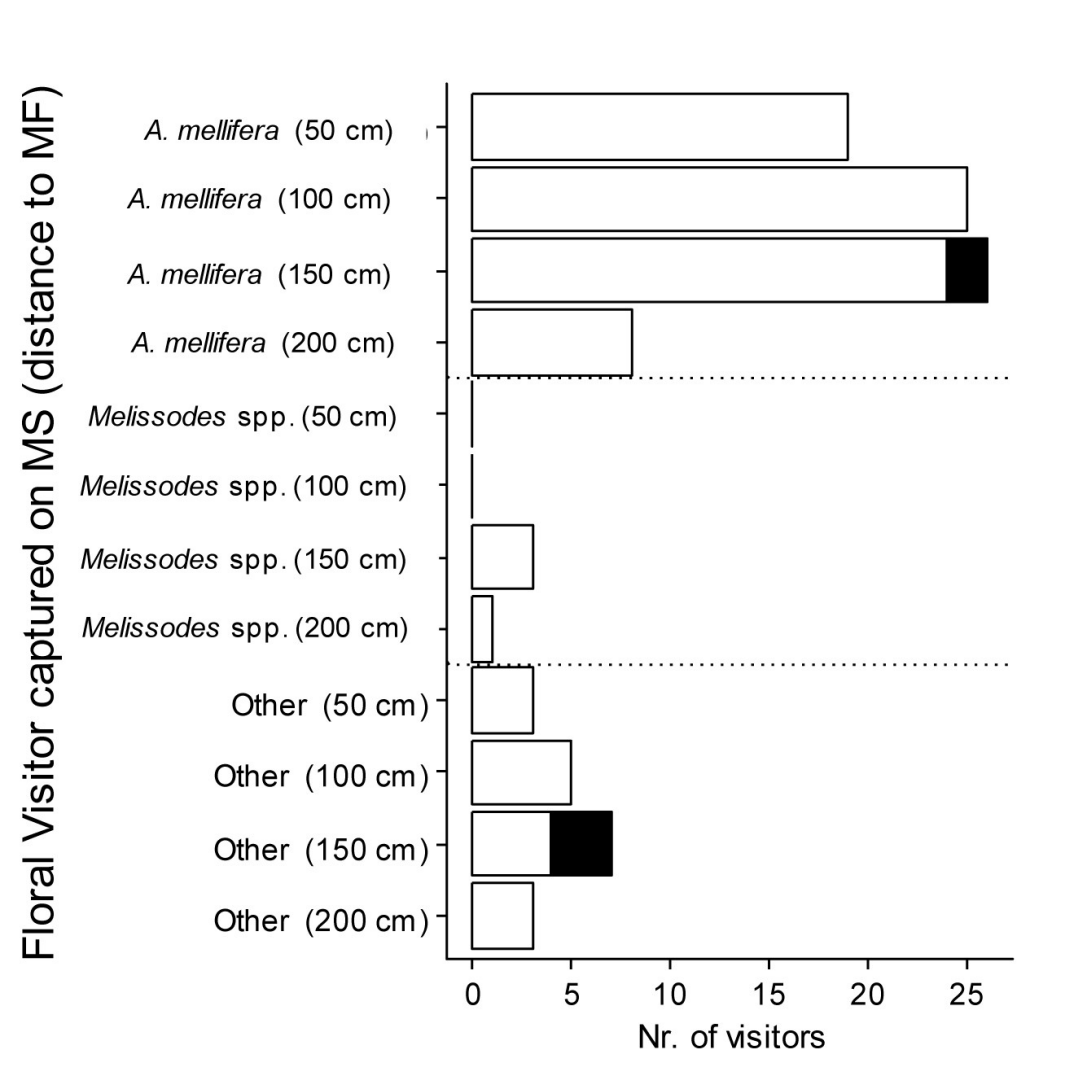
Presence of sunflower pollen on the body of main visitors on MS inflorescences. Identity and number of insect visitors captured on MS rows, at different distances to MF rows. The white bars represent the number of visitors carrying sunflower pollen grains. The black bars represent individuals without pollen grains. Most of the individuals caught had sunflower pollen on their body, regardless of the distance to pollen-donor inflorescences (*χ*_*1*_^2^ = 81, P <0.001, N = 100; Other: includes other taxa, i.e. Coleoptera, Orthoptera, Hemiptera).

Finally, despite the results described above, 95 of 100 floral visitors captured foraging on MS rows carried sunflower pollen grains on their bodies (*χ*_*1*_^2^ = 81, p < 0.001), regardless of the distance to male fertile rows (Fig 4). This observation was consistent across the taxa surveyed, the majority of which were honey bees (78% of the total visitors), consistent with their dominance in this parental line. Though most *Apis mellifera* foraged for nectar on MS inflorescences as mentioned in the previous section, 97% of the specimens captured had sunflower pollen on their bodies. The identity of the pollen obtained from the body surface matched the one obtained from MF sunflowers.

## Discussion

Our research shows that in hybrid sunflowers, which offer resources spatially separated in two highly dimorphic parental lines, honey bees and native solitary bees of the genus *Melissodes* exhibited a differential distribution between male fertile (MF) and male sterile (MS) plants. While *A*. *mellifera* was dominant on MS sunflowers, solitary pollen-specialist *Melissodes* bees represented an ample majority over other floral visitors on MF plants. Concerning their foraging behavior, most honey bees exclusively collected nectar and only a small proportion of individuals surveyed on MF rows were either gathering pollen or carried pollen loads on their corbiculae. In contrast, most *Melissodes* bees collected pollen throughout the day and visited a higher number of inflorescences per minute than honey bees, which is fully consistent with the resource exploited by each group of visitors. In regard to the transfer of pollen from MF to MS lines, both *A*. *mellifera* and *Melissodes* bees foraging on MF sunflowers showed high fidelity for this parental line and the flower constancy of *A*. *mellifera* was not affected by the interaction with a second floral visitor. Additionally, even though most honey bees foraged for nectar on MS inflorescences, the majority of them had sunflower pollen on their bodies, indicating that pollen grains ultimately are carried by social bees to the female fertile inflorescences.

### Differential spatial distribution and resource exploitation in social and solitary bees

In the agroecosystem studied, the most abundant sunflower floral visitors were honey bees *A*. *mellifera* and native solitary bees *M*. *tintinnans* and *M*. *rufithorax*. Our results are consistent with the diversity and abundance of diurnal visitors reported by Torreta and collaborators [12] for agricultural fields in the Pampean region. Both species of the genus *Melissodes* are classified as oligolectic and the pollen of sunflower represents a major component of their diet in the temperate region of Argentina [13].

The two bee populations studied, *A*. *mellifera* and *Melissodes* spp., showed a differential spatial distribution within the sunflower crop. In the seed-production cultivar evaluated, in which morphological differences between parental lines were significant, most honey bees foraged on MS and collected nectar. It is worth noting that honey bees foraging on MS rows outnumbered those on MF (9-fold increase in the density of *A*. *mellifera* foraging on MS compared to MF). These findings are in accordance with Parker’s observation [14], but differ from the densities reported in previous studies [10, 33], which did not find significant differences in the total number of honey bees between parental lines. In the agroecosystem studied, the low abundance of honey bees observed in MF lines could be determined by the reduced availability of sunflower pollen (low MF:MS rows ratio) and the low preference of honey bees to this resource [34] together with other potential pollen sources.

On the other hand, *Melissodes* spp. mostly foraged on MF lines. Indeed, honey bees were far outnumbered by *Melissodes* spp. on MF rows, even though the *Apis mellifera* population was considerably larger than the *Melissodes* population in the studied agroecosystem. On this parental line, the latter mostly gathered both nectar and pollen. This differential exploitation of floral resources can be explained by the differences between social and solitary bees. Most honey bees collect and store nectar to fulfill the colony demands, specializing in the collection of either nectar or pollen during each foraging bout [35]. In contrast, individuals of solitary bees do not exhibit such specialization in the collection of resources and such task would be accomplished most efficiently by foraging on MF sunflowers [10]. In accordance with our results, Tepedino and Parker [36] demonstrated that another oligophage species of the same genus, *Melissodes agilis*, preferred MF over MS sunflower cultivars, being male fertile inflorescences the only ones supplying both nectar and pollen.

Previous studies conducted in commercial fields in North America, where sunflowers are native, have found a great diversity of native wild bees foraging on this crop, among which *Melissodes* spp. were the most common visitors whereas honey bees were infrequent [9]. Also, they showed high per-visit pollination efficiency on a male-sterile hybrid and their visitation length was considerably longer than the one of the bee populations present in our study. However, such efficiency may be diminished if male-sterile and male-fertile lines are highly dimorphic. In addition, worldwide sunflower production relies on regions outside its native distribution [37], where pollinator diversity may be restricted to a few species and managed honey bees for pollination services are dominant. In such scenario, identifying the key functional pollinator groups in each agroecosystem and understanding their behavior can help to better assess their contribution to pollination [38, 39].

### Floral constancy and potential transfer of pollen in a dimorphic crop

Concerning the movement of bees between parental lines, both *A*. *mellifera* and *Melissodes* spp. exhibited high floral constancy when foraging in MF, reducing the potential transfer of pollen from MF to MS sunflowers. In agreement with Susic Martin and Farina [21], the reduced frequency of flights between parental lines can be a consequence of the marked dimorphism between MF and MS plants. This fidelity to one parental line observed in honey bees in the present study was not altered by interspecific interactions with a second floral visitor, contrary to Carvalheiro and collaborators [8], who found that the arrival of another bee enhanced movement of *A*. *mellifera* among inflorescences. Furthermore, our results show that the few honey bees which flew to another sunflower did not switch between parental lines, in contrast with Greenleaf and Kremen’s [11] observations. This discrepancy could be due to the different sample size, since these authors did not discriminate the identity of interacting bees, pooling 33 wild species together. Moreover. their degree of sociability might affect the interaction, in relation to the resource being foraged to meet the energetic requirements of a solitary bee, or of a colony in the case of eusocial bees. Bearing this in mind, in the present work we circumscribed to *Melissodes* and *A*. *mellifera*, both clearly contrasting in their life history.

On the other hand, in our study, all honey bees captured foraging on MS inflorescences carried sunflower pollen on their bodies. In the case of highly dimorphic hybrids where honey bees show marked fidelity to MS rows, the transfer of pollen directly by a bee moving between parental lines seems to be rare. Instead, it could be obtained from contacts with nestmates at the hive [21, 33], or by picking up on their bodies pollen previously deposited by solitary bees on an inflorescence [40]. The latter seems unlikely since few *Melissodes* bees foraged on MS plants. The former is in accordance with a previous study carried out in sunflower hybrids with high dimorphic parental lines, in which honey bees located in different areas of the colony (i.e., at the hive entrance, performing guarding tasks, or inside the hive, receiving food) exhibited pollen adhered to their bodies [21]. Thus, nestmate contacts, exclusive of social bees, could be an alternative that partially compensate the low switching frequency between parental lines mitigating a negative impact on pollination caused by large populations of pollen-specialist bees foraging mainly on MF sunflower.

### Conclusions and implications

Overall, our results show that *M*. *tintinnans* and *M*. *rufithorax* exhibit dominance in MF sunflowers, high fidelity to this parental line and are efficient pollen gatherers. Parker [14] noted that the high efficiency of oligolectic bees in collecting sunflower pollen might circumvent pollination. Moreover, sunflower reproductive success can be affected not only as a result of oligoleges depleting resources, but also due to honey bees’ preferences to collect nectar offered by MS inflorescences. On top of the differential collection of resources, the planting schemes, the size of the non-*Apis* populations [10], and the high visitation rate of native sunflower specialists, might impact crop pollination in this agroecosystem.

A thorough understanding of the different factors involved in a pollinator-dependent agricultural setting, such as the community composition of floral visitors, their foraging strategies and floral constancy and the effects of their interactions [41] is needed to assess the impact on crop yield. In particular in this sunflower agricultural setting, the presence of *M*. *tintinnans* and *M*. *rufithorax* would not contribute to sunflower pollination neither directly (due to the lack of visits to MS lines) nor indirectly (via interactions with *A*. *mellifera*). Nevertheless, the social biology of managed honey bees, which implies interactions between nestmates inside the colony, could explain the presence of pollen grains on the bodies of foragers on MS sunflower, potentially enabling pollination. Although our data are specific to sunflower hybrid seed production it is likely that a similar scenario occurs in crops with dimorphic parental lines, in which pollen is offered by male fertile plants only.

Our research aimed to understand the relationship between the pollinators in an agroecosystem that offers the resources spatially separated in two parental lines which differ morphologically. The present study did not evaluate the crop productivity directly, but this scenario, involving a crop with highly dimorphic flowers, opens the question of the relative contribution on pollination of assemblages of native solitary and introduced social bees, depending on the ecological context (see Garibaldi and collaborators [3]). In such agricultural settings, the presence of a large population of the native non-*Apis* bee (specifically, *Melissodes* spp.) would result less beneficial than expected, due to its pollen specialization and its clear parental line constancy. Additionally, the contribution of honey bees as pollinator seems to be diminished in sunflower crops with high dimorphism between parental lines due to *Apis* bees exploiting mainly MS inflorescences, reducing the occurrence of a direct transfer of pollen on the crop. The results suggest that more efforts should be done to design planting schemes that improve the efficiency of entomophilous pollination in highly dimorphic crops; moreover, since breeding programs are constantly developing high-yielding varieties.

## Supporting information

S1 Table Set of variables considered in the generalized linear model explaining the foraging behavior of *Apis mellifera* and *Melissodes* spp. (PDF)

S2 Table List of sunflower floral visitors captured on male fertile (MF) or male sterile (MS) parental lines, and subsequently identified to the lowest taxonomic level possible. (PDF)

S1 Dataset (XLSX)
