# Supplementary material for: Honey bee and native solitary bee foraging behavior in a crop with dimorphic parental lines
Source: PLoS One. 2019 Oct 11;14(10):e0223865. doi: 10.1371/journal.pone.0223865 (PMC6788694; doi:10.1371/journal.pone.0223865)
Supplement: S1 Table — (PDF) [file pone.0223865.s001.pdf]

Honey bee and native solitary bee foraging behavior in a crop with dimorphic parental lines

María Cecilia Estravis Barcala[1,2,¶]; Florencia Palottini[1,2,¶] , Walter Marcelo Farina[1,2,*].

[1]Universidad de Buenos Aires, Facultad de Ciencias Exactas y Naturales, Departamento de Biodiversidad y Biología Experimental, Laboratorio de Insectos Sociales.

[2]CONICET-Universidad de Buenos Aires, Instituto de Fisiología, Biología Molecular y Neurociencias (IFIBYNE), Buenos Aires, Argentina.

*Correspondence author

E-mail: walter@fbmc.fcen.uba.ar

[¶] These authors contributed equally to this work.

S1 Table. Set of variables considered in the generalized linear model explaining the foraging behavior of *Apis mellifera* and *Melissodes* spp.

**Section**	**Variable**	**Statistics value**	**P**
Number of inflorescences visited	Visitor x Parental line	LR= 0.4928	0.4827
	Parental line	LR= 0.01598	0.89939
	Visitor	LR= 37.5487	8.92e-10
Resource exploited	Visitor x Parental line	LR= 30.752	2.932e-08
Resource exploited on MF	Visitor x Time of day	LR=3.6081	0.0575
	Visitor	LR=37.741	8.079e-10
	Time of day	LR=50.438	1.23e-12
