# Supplementary material for: Honey bee and native solitary bee foraging behavior in a crop with dimorphic parental lines
Source: PLoS One. 2019 Oct 11;14(10):e0223865. doi: 10.1371/journal.pone.0223865 (PMC6788694; doi:10.1371/journal.pone.0223865)
Supplement: S2 Table — (PDF) [file pone.0223865.s002.pdf]

S2 Table. List of sunflower floral visitors captured on male fertile (MF) or male sterile (MS) parental lines, and subsequently identified to the lowest taxonomic level possible.

**Order**	**Family**	**Genre**	**Species**	**Total nr. of surveyed individuals on MF sunflowers**	**Total nr. of surveyed individuals on MS sunflowers**
Hymenoptera	Apidae	*Apis*	*Apis mellifera*	158	1512
		Melissodes	*Melissodes tintinnans, Melissodes rufithorax*	406 (16 males)	26 (4 males)
	Other			9	2
Coleoptera	Cantharidae	*Chauliognathus*	*Chauliognathus* sp.	137	82
	Other			10	15
Diptera				22	27
Orthoptera				8	69
Hemiptera				22	3
Neuroptera	Mantispidae			3	0
Lepidoptera				0	4
**TOTAL**				**775**	**1740**
